# Exploring the correlation between knee osteoarthritis and musculoskeletal ultrasound manifestations based on changes in traditional Chinese medical syndrome types

**DOI:** 10.1097/MD.0000000000040718

**Published:** 2024-11-29

**Authors:** Qun Qiang, Mingwang Zhou, YongXin Lv, Weibin Wang, Jia Liu, Jinqiu Wu, Xiaofei Chen, Huan Yu

**Affiliations:** a Ultrasound Medical Imaging Department, Gansu Provincial Hospital of Traditional Chinese Medicine, Lanzhou, Gansu, China; b Orthopaedics Department, Gansu Provincial Hospital of Traditional Chinese Medicine, Lanzhou, Gansu, China; c The Second Department of Articular Bone, Gansu Provincial Hospital of Traditional Chinese Medicine, Lanzhou, Gansu, China; d Radiography Department, Gansu Provincial Hospital of Traditional Chinese Medicine, Lanzhou, Gansu, China; e Department of Orthopedics, Traditional Chinese and Western Medicine Hospital of Wuhan, Tongji Medical College, Huazhong University of Science and Technology, Wuhan, Hubei, China.

**Keywords:** cartilage damage, correlation, knee osteoarthritis, musculoskeletal ultrasound, traditional Chinese medical syndrome type

## Abstract

This study explores the correlation between knee osteoarthritis and musculoskeletal ultrasound manifestations based on changes in traditional Chinese medicine (TCM) syndrome types. The study enrolled 104 patients with knee osteoarthritis admitted to the Gansu Provincial Hospital of TCM between January 2019 and January 2021. According to the principle of syndrome differentiation and treatment in TCM, the patients were divided into wind-cold-damp obstruction (n = 17), damp-heat accumulation (n = 22), qi-stagnation and blood stasis (n = 31), and liver and kidney deficiency (n = 34) types. The degrees of cartilage injury, synovial hyperplasia, synovial blood flow, and joint effusion in patients with different TCM syndrome types were compared using ultrasound. There were no significant differences in the degree of cartilage injury or synovial hyperplasia among the 4 TCM syndrome types (*P* > .05). The proportion of grade III blood flow signals in the liver and kidney deficiency group was lower than that in the damp-heat accumulation and wind-cold-damp obstruction groups, and the proportion of no blood flow and grade I blood flow signals were higher than those in the damp-heat accumulation and wind-cold-damp obstruction groups (*P* < .05). The proportion of non-joint effusion in the liver and kidney deficiency and wind-cold-damp obstruction groups was higher than that in the damp-heat accumulation and qi-stagnation and blood stasis groups (*P* < .05), and the proportion of grade I effusion in the damp-heat accumulation group was higher than that in the liver and kidney deficiency group (*P* < .05). Musculoskeletal ultrasound manifestations of knee osteoarthritis are related to TCM syndrome differentiation and classification and can provide a reference for TCM syndrome differentiation and treatment of knee osteoarthritis.

## 1. Introduction

Osteoarthritis (OA) is the most common joint disease in adults worldwide, of which knee osteoarthritis (KOA) is the most common type.^[1]^ KOA is a chronic degenerative disease characterized by degeneration and destruction of knee cartilage and subchondral bone hyperplasia. In the early stage of the disease, the knee joint is abnormally loud, sore, limp, and flaccid, and in more severe cases, will also be swollen. The cause of the disease is affected by genetics, environment, trauma, age, inflammation, and other aspects.^[2]^ The prevalence of KOA is as high as 40% in people aged 70 to 74 years, and the number of patients with KOA is gradually increasing with the aging of the population.^[3,4]^ However, there is currently no effective treatment for the etiology and underlying pathology of KOA. The 2014 International Osteoarthritis Research Institute (OARSI) guidelines for the nonsurgical treatment of knee osteoarthritis^[5]^ state that self-management and education, ground and water exercises, weight control, and strength training are the core treatments suitable for all individuals in KOA, and that no other medical treatment or physical therapy can slow the progression of the disease. In the advanced stage of KOA, patients can only undergo joint replacement surgery to improve quality of life and prolong life.

The main clinical methods for diagnosing KOA include X-ray imaging,^[6]^ arthroscopy,^[7]^ magnetic resonance imaging (MRI),^[8]^ and ultrasound.^[9]^ However, X-ray imaging focuses only on bone and ignores tissue lesions such as cartilage, synovium, joint effusion, and periarticular popliteal cysts, and cannot assess the severity of articular cartilage injury in KOA. AS has limited clinical application because of its aggressiveness (it can only be used for diagnosis during surgical treatment). Although MRI is noninvasive and has good imaging results, its application scope is limited owing to its high cost, contraindications, and false-negative rate. High-frequency ultrasound (HFUS) is a noninvasive examination that is easy to perform and inexpensive, has been widely used in the diagnosis of joint diseases, uses high-frequency probes to examine muscles, bones, joints, and other parts, and can be used to observe articular cartilage lesions and blood circulation around the joints to diagnose KOA.^[10]^ HFUS has a high resolution of soft tissues, and some studies have confirmed its good application effect in the evaluation of cartilage injuries in various subregions of the knee joint in patients with KOA using the double-scoring method.^[11]^

For a long time, traditional Chinese medicine (TCM) has accumulated experience in the prevention and treatment of KOA; however, at present, there are many types of TCM syndrome differentiation and different standards in KOA, which has brought considerable difficulty to clinicians. According to TCM theory, knee osteoarthritis has the pathogenic characteristics of “deficiency, evil, and stasis.”^[12]^ TCM has a long history of treating KOA and can provide new ideas for the clinical diagnosis and treatment of KOA. However, at present, there is no unified standard for syndrome differentiation and classification of this disease, which inevitably affects the treatment efficacy of TCM over time.^[13]^ Musculoskeletal ultrasound can provide a new perspective for syndrome differentiation and classification in TCM and is an extension of TCM inspection. This study aimed to explore the correlation between the ultrasonic features of cartilage injury in KOA patients and TCM syndrome differentiation and treatment, hoping to improve the accuracy of the clinical diagnosis of KOA so that patients can receive timely and effective treatment, and thus improve their quality of life.

## 2. Materials and methods

### 2.1. Participants

The study included 104 patients with KOA who were admitted to the Department of Joint and Orthopedics of Gansu Provincial Hospital of TCM between January 2019 and January 2021. The cohort included 44 males and 60 females, aged 40 to 70 years old, with an average age of (56.7 ± 10.52) years, a body mass index (BMI) of 20 to 28 kg/m^2^, and an average BMI of (23.19 ± 2.60) kg/m^2^. The duration of the disease ranged from 6 months to 7 years, with an average duration of (4.23 ± 3.23) years. According to the principle of syndrome differentiation and treatment of TCM in the Guidelines for the Diagnosis and Treatment of Knee Osteoarthritis with Integrated traditional Chinese and Western Medicine, the patients were divided into the wind-cold-damp obstruction (n = 17), damp-heat accumulation (n = 22), qi-stagnation and blood stasis (n = 31), and liver and kidney deficiency (n = 34) types. This study was approved by the Medical Ethics Committee of Gansu Provincial Hospital of TCM. The patients and their families provided written informed consent.

The inclusion criteria were: patients met the diagnostic criteria for primary KOA in the “2018 Guidelines for the Diagnosis and Treatment of Osteoarthritis” proposed by the Orthopaedic Branch of the Chinese Medical Association.^[14]^ Patients had complete clinical data, and underwent musculoskeletal ultrasonography.

The exclusion criteria were as follows: acute traumatic osteochondral injury; local knee surgery or obvious scarring from trauma; inability to flex the joints and severe joint deformities; and severe psychiatric illness preventing participation in the study.

### 2.2. Instruments and methods

Ultrasonography was performed using a Samsung RS80A ultrasound diagnostic instrument (Shanghai Huanxi Medical Equipment Co., Ltd.) with an L3-12A MHz linear array probe. During detection, the patient was first placed in the supine position with the knee straightened (0°). Transverse and longitudinal scans were performed on the inferior border of the patella and the popliteal fossa to examine the medial femoral intercondylar cartilage (FMC) and lateral femoral intercondylar cartilages (FLC). The knee was then flexed at 90° and the probe was placed in front of the distal trochlear joint while the distal trochlear cartilage was carefully detected. While scanning the inspection area, the operator performed a lateral scan followed by a longitudinal scan. The operator also kept the ultrasound probe perpendicular to the examination area to ensure a high resolution of the ultrasound image.

**Figure 1.**
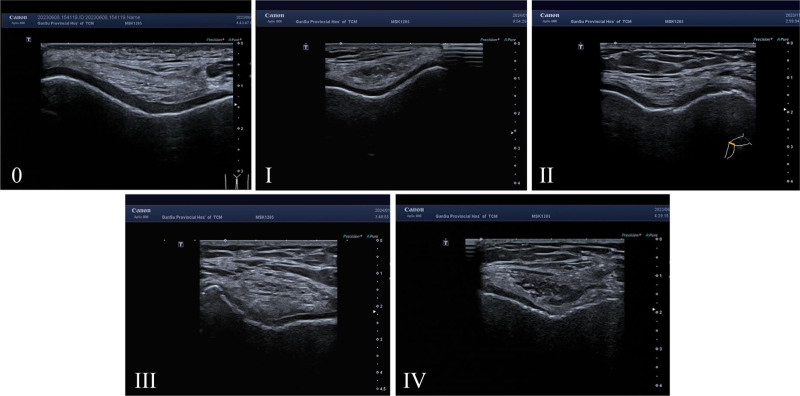
Ultrasound image characteristics of grades 0 to IV cartilage injury.

Regarding the lesion grade, the following grading criteria were used for cartilage injury^[15]^ (Fig. 1): grade 0, normal, the cartilage surface and subchondral bone lines are smooth, continuous, clear, and sharp, and the low-echo cords are uniform; grade I, no obvious degenerative changes, cartilage surface is not sharp; grade II, degenerative cartilage surface rough, locally raised or slightly thinned; grade III, degenerative cartilage is thinner, and the echo of the subchondral bone line is enhanced and irregular; and grade IV, complete loss of degenerative cartilage layer, exposure of subchondral bone, continuous or discontinuous subchondral bone line. The cartilage of the distal femur of each knee joint is divided into 3 regions: trochlear femoral, medial femoral condyle, and lateral femoral condyle. The stage of cartilage injury in this region is determined by the stage of the most severe cartilage injury. Grading criteria for synovial hyperplasia^[16]^ were as follows: grade 0, no synovial hyperplasia; grade I, synovial hyperplasia not exceeding the line of the highest point of the bone surface; grade II, synovial hyperplasia beyond the highest point of the bone surface and no more than the backbone; and grade III, exceeding the highest point of the bone surface. Synovial blood flow grading criteria were as follows^[17]^: grade 0, no flow signal; grade I, single blood flow signal; grade II, <1/2 area of the fusion flow signal; and grade III, >1/2 area of the fusion flow signal. The following criteria were used for grading joint effusions^[18]^: grade 0, no effusion; grade I, effusion thickness of 4 to 5 mm; grade II, effusion thickness of 5 to 10 mm; and grade III, effusion thickness of more than 10 mm.

### 2.3. Statistical methods

SPSS version 22.0 (Chicago) statistical software was used for the data analysis. Kolmogorov–Smirnov and Levene tests were used to detect the normality and variance homogeneity of the measurement data, respectively. Differences in the measurement data were compared using the *t*-test, and ANOVA was applied for comparison between multiple groups. Count data were compared using the chi-squared test or anecdotal sum test. Differences were considered statistically significant at *P* < .05.

## 3. Results

**Figure 2.**
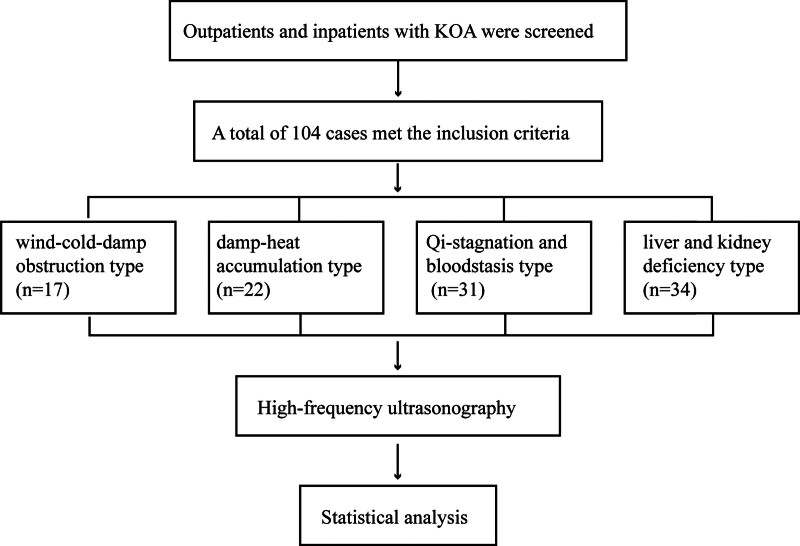
Flow chart of the study participants.

A total of 104 patients with KOA were included in this study, all of whom completed the study and were included in the statistical analyses (Fig. 2).

### 3.1. Comparison of basic data of patients with different TCM syndrome differentiation types

Among the 104 patients, 17 had wind-cold-damp obstruction, 22 had damp-heat accumulation, 31 had qi-stagnation and blood stasis, and 34 had liver and kidney deficiency. The disease course was significantly different among the 4 groups (*P* < .05), whereas there were no statistically significant differences in age, height, weight, or BMI between the 4 groups (*P* > .05), as shown in Table 1.

**Table 1:** Comparison of basic data of patients with different TCM syndrome differentiation types.

Variables	Wind-cold-damp obstruction type	Damp-heat accumulation type	Qi-stagnation and blood stasis type	Liver and kidney deficiency type	*P*-value
Age (yr)	58.4 ± 5.7	59.3 ± 7.8	56.9 ± 8.5	57.8 ± 7.9	.885
Height (cm)	158.1 ± 5.7	161.1 ± 6.3	160.0 ± 4.1	159.2 ± 6.5	.182
Weight (kg)	66.5 ± 5.8	66.5 ± 5.8	66.3 ± 7.0	66.4 ± 6.1	.573
BMI (kg/m^2^)	25.7 ± 3.2	26.4 ± 2.7	25.6 ± 3.0	25.4 ± 2.9	.672
Duration of disease (yr)	4.52 ± 1.08	3.55 ± 1.09	3.54 ± 1.08	4.55 ± 1.10	.034

### 3.2. Comparison of the degree of cartilage damage in patients with different TCM syndrome differentiation types

The results showed no statistically significant differences in the degree of cartilage injury among patients with the 4 TCM syndrome types (*P* > .05). In patients with grade III positivity, cartilage changes were greater in the liver and kidney deficiency types than in the wind-cold-damp obstruction and damp-heat accumulation types (*P* < .05), and there was no significant difference among the other syndrome types (*P* > .05) (Table 2).

**Table 2:** Comparison of the degree of cartilage injury in patients with different TCM syndrome differentiation types.

Syndrome differentiation of TCM	0	I	II	III	IV
Wind-cold-damp obstruction type	8 (47.06%)	4 (23.53%)	3 (17.65%)	2 (11.76%)	0 (0)
Damp-heat accumulation type	11 (50.00%)	5 (22.73%)	4 (18.18%)	2 (9.09%)	0 (0)
Qi-stagnation and blood stasis type	2 (6.45%)	2 (6.45%)	4 (12.90%)	16 (51.61%)	7 (22.58%)
Liver and kidney deficiency type	3 (8.82%)	2 (5.88%)	4 (11.76%)	17 (50.00%)*,†	8 (23.53%)
χ^2^ value	0.681				
*P*-value	.771				

Abbreviation: TCM = traditional Chinese medicine.

* Compared with the wind-cold-damp obstruction type, *P* < .05.

† Compared with the damp-heat accumulation type, *P* < .05.

### 3.3. Comparison of ultrasound synovial hyperplasia in patients with different TCM syndrome differentiation types

The results showed no statistically significant difference in the degree of synovial hyperplasia among patients with the 4 TCM syndrome types (*P* > .05) (Table 3).

**Table 3:** Comparison of the degree of synovial hyperplasia in patients with different TCM syndrome differentiation types.

Syndrome differentiation of TCM	0	I	II	III
Wind-cold-damp obstruction type	5 (29.41%)	6 (35.29%)	5 (29.41%)	2 (11.76%)
Damp-heat accumulation type	9 (40.91%)	5 (22.73%)	5 (22.73%)	3 (13.64%)
Qi-stagnation and blood stasis type	8 (25.81%)	6 (19.35%)	9 (29.03%)	8 (25.81%)
Liver and kidney deficiency type	7 (20.59%)	5 (14.71%)	10 (29.41%)	12 (35.29%)
χ^2^ value	0.275			
*P*-value	.871			

Abbreviation: TCM = traditional Chinese medicine.

### 3.4. Comparison of synovial blood flow signals by ultrasound of various TCM syndrome types

There were statistically significant differences in the synovial blood flow signals among the 4 syndrome types (*P* < .05) and in the proportion of grade III disease. The blood flow signals in the liver and kidney deficiency group were lower than those in the damp-heat accumulation and wind-cold-damp obstruction groups, and the proportions of non-blood flow signals and grade I blood flow signals were higher than those in the damp-heat accumulation and wind-cold-damp obstruction groups, as shown in Table 4.

**Table 4:** Comparison of synovial blood flow signals in patients with different TCM syndrome differentiation types.

TCM syndrome differentiation	0	I	II	III
Wind-cold-damp obstruction type	3 (17.65%)	6 (35.29%)	4 (23.53%)	4 (23.52%)
Damp-heat accumulation type	3 (13.64%)	7 (31.82%)	8 (36.36%)	4 (18.28%)
Qi-stagnation and blood stasis type	7 (22.58%)	10 (32.26%)	11 (35.48%)	3 (9.68%)
Liver and kidney deficiency type	7 (20.59%)*,†	13 (38.24%)*,†	10 (29.41%)	4 (11.76%)*,†
χ^2^ value	13.078			
*P*-value	.001			

Abbreviation: TCM = traditional Chinese medicine.

* Compared with the wind-cold-damp obstruction type, *P* < .05.

† Compared with the damp-heat accumulation type, *P* < .05.

### 3.5. Comparison of joint effusion under ultrasound of different TCM syndrome types

There were statistically significant differences in joint effusion among the 4 syndrome types (*P* < .05), and the proportion of no joint effusion in the liver and kidney deficiency and wind-cold-damp obstruction groups was higher than that in the damp-heat accumulation and the qi-stagnation and blood stasis groups (*P* < .05). The proportion of grade I effusion in the damp-heat accumulation group was higher than that in the liver and kidney deficiency group (*P* < .05), as shown in Table 5.

**Table 5:** Comparison of joint effusion in patients with different TCM syndrome differentiation types.

Syndrome differentiation of TCM	0	I	II	III
Wind-cold-damp obstruction type	3 (17.65%)*,†	4 (23.52%)	6 (35.29%)	4 (23.52%)
Damp-heat accumulation type	3 (13.64%)	10 (45.45%)‡	7 (31.82%)	2 (9.10%)
Qi-stagnation and blood stasis type	5 (16.13%)	10 (32.26%)	11 (35.48%)	5 (16.13%)
Liver and kidney deficiency type	6 (17.65%)*,†	14 (41.18%)	10 (29.41%)	4 (11.76%)
χ^2^ value	18.103			
*P*-value	.000			

Abbreviation: TCM = traditional Chinese medicine.

* Compared with the damp-heat accumulation group, *P* < .05.

† Compared with the qi-stagnation and blood stasis group, *P* < .05.

‡ Compared with the liver and kidney deficiency group, *P* < .05.

## 4. Discussion

Most patients with KOA have irreversible degeneration of the articular cartilage before the typical radiographic changes.^[2]^ At present, arthroscopy is the gold standard for diagnosing KOA; however, because it is a traumatic examination, complications can easily increase. MRI scans are time-consuming and expensive, and are not conducive to multiple follow-ups. Musculoskeletal ultrasonography is better than MRI for detecting synovial blood flow and is more cost-effective than radiography for observing joint effusion, synovitis, bone destruction, and erosion.

Musculoskeletal ultrasound can clearly display the synovium, which in hyperplasia is mainly convex towards the joint cavity and isonic or hypoechoic, and the sonogram of color Doppler imaging technology shows stellate blood flow signals, which can better evaluate the state of illness.^[19]^ On musculoskeletal ultrasound, the capsule of the popliteal cyst appears smooth and irregularly echogenic.^[20]^ Musculoskeletal ultrasound has been widely used in the diagnosis, treatment and efficacy of KOA, and can provide a basis for syndrome differentiation and classification of KOA.^[21]^ Studies have shown that female sex, age ≥ 60 years, BMI ≥ kg/m^2^, and joint weight-bearing are risk factors for KOA in Chinese groups.^[22]^ The patient cohort in this study generally met the characteristics of the disease; there were more females than males, obesity, and a predominance of middle-aged and elderly people. However, the results showed that age and BMI could not be used as bases for syndrome differentiation in TCM for KOA. In this study, TCM syndrome type analysis of patients with KOA showed that liver and kidney deficiency syndromes were the main syndrome type of the disease. With increasing age, the human kidney essence is gradually depleted, the muscles and bones change from prosperity to decline, and liver and kidney deficiencies play an important role in the occurrence and development of KOA, which is in line with physiological changes in human kidney essence, qi, and blood.^[23]^ The results of this study showed that the disease course was different in each of the 4 syndrome types, and was longer in the liver and kidney deficiency and wind-cold-damp obstruction groups than in the other 2 groups, suggesting that the disease course of KOA was reflected in the pattern from qi-stagnation and blood stasis to wind-cold-damp obstruction to damp-heat accumulation and liver and kidney deficiency.

Most patients with KOA have varying degrees of synovial thickening; however, a few have active synovitis, which is consistent with the basic pathology of KOA.^[24]^ Yu^[25]^ found differences in synovial thickness among the 4 syndrome types, with the thickest synovial thickness in patients with liver and kidney deficiency and the thinnest synovial thickness in patients with damp-heat accumulation, indicating that synovial thickness can provide a basis for the differentiating between the liver and kidney deficiency and damp-heat accumulation types. The results of this study showed no significant differences in the synovial thickness among the 4 syndrome types. Zheng^[26]^ found significant differences in the degree of cartilage damage under arthroscopy among the syndrome types; although different TCM syndrome types were used, the conclusion that there were differences in pathological manifestations among the syndrome types was supported to a certain extent. The results of this study showed no statistically significant difference in cartilage injury among the 4 syndrome types, which is consistent with the results of Yu study.

Blood flow signals reflect tissue inflammation. In the present study, the proportion of patients with grade III disease was calculated. Blood flow signals in the liver and kidney deficiency group were lower than those in the damp-heat accumulation and wind-cold-damp obstruction groups, and the proportions of no blood flow signal and grade I blood flow signal were higher than those in the damp-heat accumulation and wind-cold-dampness groups. From the perspective of syndrome differentiation in TCM, the main manifestations of wind-cold-dampness syndrome are joint pain, local burning, and morning stiffness, and the degree of synovitis is higher in these patients.^[27]^ However, joint swelling and pain are not obvious in patients with liver and kidney insufficiency syndrome, and the degree of inflammation is low. The TCM theory states that if the liver and kidney are insufficient, the muscles and bones are not nourished, and kidney deficiency is the main mechanism for the formation of osteophytes.^[28]^ The results of this study also showed that the proportion of no joint effusion was higher in the liver and kidney deficiency and wind-cold-damp obstruction groups than in the damp-heat accumulation and qi-stagnation and blood stasis groups. Joint effusion is also an inflammatory reactant, and patients with liver and kidney deficiency syndrome have phlegm, dampness, stasis, toxicity, and obstruction. Effusion accumulates over time, and the muscles and bones lose nourishment, so the incidence of joint effusion is higher than that of other syndrome types, which is consistent with the results of Chen et al.^[29]^ In summary, musculoskeletal ultrasound manifestations of KOA are related to TCM syndrome differentiation and classification, and can provide a reference for TCM syndrome differentiation and treatment of KOA.

This study had the following limitations: it was a cross-sectional study and did not assess the long-term risk of TCM syndrome differentiation and KOA development. However, although ultrasound has a high resolution for local soft tissues, it lacks utility for evaluating overall mechanical and microscopic biochemical changes in the knee joint, and has certain blind spots. Our results are also not representative of other ethnic groups and races, as this study only evaluated patients with KOA in western China, and further research should be conducted on the syndrome differentiation and characterization of KOA ultrasound images in patients of different ethnicities, races, and age groups. In addition, the relatively small sample size of this study limits the reliability of the data. Therefore, cohort studies with larger sample sizes, especially prospective cohort studies, should be conducted in the future to evaluate the correlation between different types of TCM syndrome differentiation and musculoskeletal ultrasound findings.

## 5. Conclusion

The musculoskeletal ultrasound manifestations of KOA are related to syndrome differentiation and TCM classification. In patients with grade III positivity, the cartilage changes in the liver and kidney deficiency types were greater than those in the wind-cold-damp obstruction and damp-heat accumulation types. The proportion of patients with synovial grade III blood flow signals was lower in the liver and kidney deficiency types than in the damp-heat accumulation and wind-cold-damp obstruction types, while the proportions of synovial non-blood flow signals and grade I blood flow signals were higher than those in the damp-heat accumulation and wind-cold-damp obstruction types. The proportion of patients with no joint effusion was higher in the liver and kidney deficiency type and wind-cold-damp obstruction group than in the damp-heat accumulation and qi-stagnation and blood stasis types. Ultrasonography provides new and important information for the objective study of TCM syndrome differentiation and classification, and is worthy of further in-depth study.

## Acknowledgments

This work was supported by the Gansu Provincial Science and Technology Plan Project-Clinical Research Center for Osteoarticular Degenerative Diseases, Research on Diagnosis and Clinical Evaluation of Primary Knee Osteoarthritis (Project No. 18JR2FA009), the Natural Science Foundation of Gansu Province (Project No. 22JR5RA627), and Key Research and Development Program of Gansu Province (Project No. 22YF7FA103).

## Author contributions

**Conceptualization:** Mingwang Zhou, YongXin Lv, Weibin Wang, Jia Liu.

**Formal analysis:** Jinqiu Wu.

**Methodology:** Xiaofei Chen, Huan Yu.

**Writing – original draft:** Qun Qiang.

**Writing – review & editing:** Qun Qiang, Mingwang Zhou.
